# RAC1 is involved in uterine myometrium contraction in the inflammation-associated preterm birth

**DOI:** 10.1530/REP-21-0186

**Published:** 2022-08-26

**Authors:** Min Diao, Jin Zhou, Yunkai Tao, Zhaoyang Hu, Xuemei Lin

**Affiliations:** 1 Department of Anesthesiology, West China Second University Hospital, Sichuan University, Chengdu, Sichuan, China; 2 Key Laboratory of Birth Defects and Related Diseases of Women and Children, Sichuan University, Ministry of Education, Chengdu, Sichuan, China; 3 Laboratory of Anesthesia and Critical Care Medicine, National-Local Joint Engineering Research Centre of Translational Medicine of Anesthesiology, Department of Anesthesiology, West China Hospital, Sichuan University, Chengdu, Sichuan, China

## Abstract

**In brief:**

Various etiologies can cause uterine myometrium contraction, which leads to preterm birth. This study demonstrates a new functional relationship between the Ras-related C3 botulinum toxin substrate 1 (RAC1) and uterine myometrium contraction in preterm birth.

**Abstract:**

Preterm birth (PTB) is a public health issue. The World Health Organization has recommended the use of tocolytic treatment to inhibit preterm labour and improve pregnancy outcomes. Intrauterine inflammation is associated with preterm birth. RAC1 can modulate inflammation in different experimental settings. In the current study, we explored whether RAC1 can modulate spontaneous uterine myometrium contraction in a mouse model of lipopolysaccharide (LPS)-induced intrauterine inflammation. Subsequently, we recorded uterine myometrium contraction and examined uterine *Rac1* expression in a mouse model of preterm birth and a case in pregnant women by Western blotting analysis. We also measured progesterone levels in the blood serum of mice. Murine myometrium was obtained 12 h post LPS treatment. Human myometrium was obtained at the time of caesarean section. We found that in the LPS-treated group of mice, uterine myometrium contraction was enhanced, protein levels and activation of RAC1 were increased and serum progesterone levels were decreased. The protein levels of RAC1 were also increased in preterm birth and in pregnant women. NSC23766, a RAC1 inhibitor, attenuated uterine myometrium contraction and diminished RAC1 activation and COX-2 expression. Furthermore, silencing of RAC1 suppressed cell contraction and COX-2 expression* in vitro*. In conclusion, our results suggested that RAC1 may play an important role in modulating uterine myometrium contraction. Consequently, intervening with RAC1 represents a novel strategy for the treatment of preterm birth.

## Introduction

Preterm birth (PTB) is a life-threatening neonatal disease. One survey found that the worldwide rate of PTB in 2014 was 10.6% of all live births, which is higher than the rate in 2010 (Chawanpaiboon *et al.* 2019). It is now estimated that PTB complications are a leading cause of death under 5 years of age, along with infectious diseases (WHO 2020). PTB can cause not only neonatal mortality but also several types of neonatal morbidity. The major complications of PTB are adverse neurological outcomes, diseases of immature organ systems and lifelong high risk of chronic disease (Mwaniki *et al.* 2012). The burden of preterm birth is immense due to the high economic and social costs.

Studies of the causes of PTB have shown that numerous maternal factors, including maternal age, multiple pregnancy, maternal infections, maternal tobacco use and maternal alcohol or substance abuse, are involved (Muglia & Katz 2010, Ferré *et al.* 2016). In essence, these underlying factors cause the uterus to change from a quiescent to a contractile state (Blencowe *et al.* 2012). The molecular and cellular mechanisms underlying myometrial contraction vary. Calcium is a key regulator of the contractile machinery of smooth muscle tissue. Substantial progress has been made in understanding the molecular and cellular mechanisms underlying the modulation of intracellular Ca^2+^ levels, including mechanisms related to G-protein coupled receptor/IP3 (Phaneuf *et al.* 1993, Aguilar & Mitchell 2010) and the regulation of various Ca^2+^ channels (Sanborn 2000, Hösli *et al.* 2014) and Ca^2+^-sensitive K+ channels (Ledoux *et al.* 2006). In addition to those mentioned previously, other mechanisms are involved in contraction, including calcium sensitization (Riley *et al.* 2005), myosin light chain phosphatase activity (Hartshorne *et al.* 2004) and prostaglandin, oxytocin, vasopressin and neurotransmitter production (Abdalla *et al.* 2004). Suppression of uterine activity has currently received much attention in studies on PTB treatment and prevention. At present, there are many well-characterized and clinically used agonists and antagonists of many uterine smooth muscle pathways, such as Ca^2+^ channel blockers, oxytocin, atosiban and progesterone (Garfield & Chin 2020). To overcome the limited efficacy of existing therapeutics, further research is being performed to investigate the mechanisms underlying contraction and to identify drug targets within that mechanism.

The Rho GTPase family consists of at least 20 members, including inactive GDP-bound forms and active GTP-bound forms. Rho GTPases play significant roles in modulating myometrial contractility. Accumulating evidence has indicated that RhoA, a member of the Rho GTPase family, is involved in calcium sensitization. The RhoA/ROCK signalling pathway is associated with the inhibition of myosin light chain phosphatase (MLCP), which regulates uterine smooth muscle contraction by non-calcium-dependent mechanisms (O’Brien *et al.* 2008, Chen *et al.* 2019). In contrast to RhoA, the function of Ras-related C3 botulinum toxin substrate 1 (RAC1), another member of the Rho GTPase family, in the regulation of uterine smooth muscle contraction has not been well studied.

As an intracellular signalling molecule activated by extracellular stimuli, RAC1 not only is implicated in the pathogenesis of cardiac hypertrophy (Satoh *et al.* 2006) and hypertension (Hassanain *et al.* 2007) but also regulates bronchoconstriction (Andre-Gregoire *et al.* 2018). RAC1 plays a crucial role in smooth muscle cell (SMC) contraction. Recently, evidence from previous studies has shown that the activation of RAC1 induces airway SMC contraction and results in airway hyperresponsiveness associated with asthma (Andre-Gregoire *et al.* 2018, Kai *et al.* 2019); additionally, the activation of RAC1 regulates the vascular SMC contraction involved in the pathogenesis of hypertension (André *et al.* 2014). RAC1 has long been recognized to play an essential role in the regulation of intracellular Ca^2+^ (Zhang *et al.* 2016, Andre-Gregoire *et al.* 2018) and the Ca^2+^‐sensitive MLCP pathway (Shibata *et al.* 2015, Kai *et al.* 2019). There are few reports focusing on the potential link between RAC1 and uterine SMC contraction, and how RAC1 regulates uterine SMC contraction remains unknown and warrants investigation.

In the present study, we investigated the role of RAC1 in uterine myometrium contraction. We speculated that RAC1 might regulate uterine function. To elucidate the roles of RAC1 signalling in uterine SMC contraction, we administered intrauterine lipopolysaccharide (LPS) treatment to cause PTB in mice. *Rac1* expression in the myometrium was assessed, and spontaneous uterine myometrium contraction was recorded. Then, we examined whether i.p. administration of NSC23766, RAC1 inhibitor, had any inhibitory effects on spontaneous uterine myometrium contraction.

## Materials and methods

### Animals

Seven- to eight-week-old CD-1® (ICR) mice were purchased from Beijing Vital River Laboratory Animal Technology Co., Ltd. and housed in a standard environment with constant humidity (55%), regulated temperature (21 ± 2°C) and a 12 h light:12 h darkness cycle in the Laboratory Animal Centre of West China Second University Hospital, Sichuan University. This study was approved by the Sichuan University Committee on Animal Research (2018059), and all animal procedures were performed in accordance with the National Institutes of Health guidelines for the care and use of laboratory animals. Timed pregnancies were obtained by mating one male mouse with two nulliparous females (vaginal plug = day 1 of pregnancy).

### Surgical procedures and treatment

We used a model of preterm birth as described in an established protocol (Burd *et al.* 2010). Mice were anaesthetized with 3% isoflurane inhalation at 8:00 h on gestational day 16 (GD16). The uterine horns were exposed by careful dissection through a miniature incision in the lower abdomen. Then, either LPS (from *Escherichia coli* O55:B5, L2880, Sigma–Aldrich) dissolved in normal saline solution (concentration 1 µg/μL, 150 μg LPS/dam, *n*  = 16) or an equal volume of saline (control, *n*  = 22) was injected into the uterus between the two gestational sacs closest to the cervix; injection into the gestational sacs was avoided. To investigate whether RAC1 has an effect on preterm delivery, the RAC1 inhibitor NSC23766 (5 mg/kg, 2161, Tocris Bioscience, Bristol, United Kingdom) was intraperitoneally injected into mice 0.5 h before and after the intrauterine injection of LPS or saline (LPS + NSC23766, *n*  = 15; control + NSC23766, *n*  = 15) (Schematic diagram shown in Supplementary Fig. 1, see section on supplementary materials given at the end of this article). The dosage of NSC23766 was chosen based on our preliminary experiments and a previous study (Wang *et al.* 2016). Pregnant mice were euthanized at 12 h after LPS injection. Any signs of the onset of labour, including vaginal bleeding, intrauterine fetal displacement (during repeat laparotomy) and delivery of at least one pup, were recorded from the time of LPS injection to tissue harvest. If all the pups of the pregnant mice were born before 12 h post LPS injection, uterine tissue and maternal blood were not collected. To observe pregnancy outcomes with LPS and NSC23766 exposure, a second set of mice (*n*  = 5 per group) was allowed to deliver. The timing of preterm delivery post LPS treatment was recorded. The average gestational duration from LPS treatment to delivery was observed.

### Tissue collection

#### Mouse tissue

In the present study, mice were anaesthetized again at 12 h after LPS administration to collect blood for the detection of serum progesterone levels. Then, the mice were humanely killed, and the right side of the uterus was harvested from each mouse. The uterus was excised and opened longitudinally, and adipose tissue, fetuses, placentas and fetal membranes were gently removed from the uterus. Then, the endometrium was scraped carefully with a surgical blade and removed using a cotton-wool bud. Only the myometrial layer was used as a sample as much as possible. Under the microscope, the myometrial layer had many longitudinal stripes. All myometrium samples were washed with cold PBS and then 3 sections were cut at random. The sections were immediately stored at −80°C for Western blotting analysis, fixed in 4% paraformaldehyde for paraffin sectioning or placed into ice-cold PBS for spontaneous uterine myometrium contraction recording.

#### Human myometrial tissue

After receiving written informed consent, we enrolled four term pregnant women who were not in labour and undergoing elective caesarean section and four preterm pregnant women (<37 weeks) with contraction pain who were undergoing emergency caesarean section. None of the pregnant women had obstetrical complications or medical and surgical complications, including gestational hypertension and gestational diabetes. All the women in labour were classified as American Society of Anaesthesiologists status I or II. The enrollment procedures were reviewed by the local Ethics Committee of West China Second University (2012001) and followed the ethical standards of the Declaration of Helsinki. As explained in a previous study (Li *et al.* 2016), biopsies of myometrium were excised from the middle portion of upper edge of the incision line in the lower uterine segment at caesarean section. The amnion and chorion laeve were separated. The collected samples were immediately placed in liquid nitrogen and transferred to the labour atory for Western blotting.

### Measurement of serum progesterone levels

The blood from pregnant mice was collected at 12 h after intrauterine injection. Serum progesterone concentrations were measured with an ELISA kit (EK7004, Boster Biological Technology, Pleasanton CA, USA)) according to protocols provided by the manufacturers.

### Spontaneous uterine myometrium contraction recording

Mouse uterine tissue samples were placed in ice-cold carbogenated (95% O_2_-5% CO_2_) PBS, and the adipose tissue, placenta and uterine connective tissue were removed. Longitudinal myometrial strips (0.5 cm) were collected and hung in an organ bath system filled with carbogenated (95% O_2_, 5% CO_2_) Krebs solution (119 mM NaCl, 4.7 mM KCl, 25 mM NaHCO_3_, 1.2 mM KH_2_PO_4_, 2.5 mM CaCl_2_, 1.5 mM MgSO_4_, 11 mM glucose) at 37°C. As described by Brown *et al.* (2007), one end of the myometrial strip was held in a fixed position by a hook, and the other end was attached to a force transducer via an adjustable metal hook. Then, the myometrial strip was equilibrated for 30 min with a basal tension of approximately 2 g, at which point, the strip was approximately two times its original length. After regular spontaneous myometrial contractility was observed, contractile activity data were recorded for a 10-min period and analysed using data software (BL-420s Biological Data Acquisition & Analysis System, Chengdu TME Technology Co., Ltd., China).

In our experiment, we used eight parameters to assess spontaneous myometrial contractility during the 10-min period of rhythmic contraction. Contractility parameters were frequency (/min), contractile amplitude (g), area under the curve (g*s), contraction tension (g), mean tension (g), duration (s), time between contractions (s) and ratio of contraction. Some parameters, such as contractile amplitudes (g), area under the curve (g*s), contraction tension (g), mean tension (g), duration (s) and time between contractions (s), were recorded by data software. Other parameters were calculated. Frequency was defined as the total number of contractile waveforms divided by the total duration of the period assessed. The ratio of contraction was defined as the duration of the contractile waveform divided by the time from the start of one contraction wave to the start of the next.

### Tissue processing for histological evaluation

Sections of myometrial strips were stained with haematoxylin–eosin (H&E) and photographed under an inverted microscope (Olympus A/S) (magnification, 10× and 40×). Briefly, myometrial inflammatory infiltration was graded (Schafer *et al.* 2018) by a pathologist according to severity (0 = no damage, 1= mild, 2 = moderate, and 3 = marked).

### Culture of mouse uterine smooth muscle cells and Rac1 ShRNA transfection

Mouse uterine smooth muscle cells (USMCs) were isolated by enzymatic dispersion. The uterine tissues of pregnant mice were isolated under sterile conditions, and adipose tissue, fetuses, placentas and fetal membranes were gently removed from the uterus in PBS containing 100 U/mL penicillin and 100 μg/mL streptomycin. The smooth muscle layers were cut into pieces and incubated in Dulbecco’s modified Eagle’s medium (DMEM, Gibco) containing 2 mg/mL collagenase type II (C2-22, Sigma–Aldrich) at 37°C for 60 min. After mixing and centrifugation, the cell deposit was resuspended in DMEM containing 10% fetal bovine serum (FBS, Gibco, Thermo Fisher Scientific, Inc.), 100 U/mL penicillin and 100 μg/mL streptomycin. The cells were then plated into 10 cm^2^ flasks and kept at 37°C in a 5% CO_2_-95% air humidified atmosphere until confluent. The experiments were performed on cells in passage 2.

To specifically interfere with *Rac1* expression, RNA interference (RNAi) was used in the present study. Adenovirus-mediated short hairpin (ShRNA) targeting *Rac1* plasmid (Sh-*Rac1*, top strand: GCAGACAGACGTGTTCTTAATTTGCT and bottom strand: CAGACAGACGTGTTCTTAATTTGCT) and the negative control (Sh-NC, top strand: TTCTCCGAACGTGTCACGTAA and bottom strand: AAAAATTCTCCGAACGTGTCA) were commercially procured from Hanbio Tech (Shanghai, China). *Rac1*-specific or control shRNA was transfected according to the manufacturer’s instructions. Immunoblotting analysis was used to examine *Rac1* silencing 48 h after transfection. Additionally, cells were treated with 100 ng/mL LPS for 24 h, which could trigger strong immune‑inflammatory responses, whereas cells treated with PBS served as controls.

### Cell contraction assay

A collagen contractility assay was used to evaluate the contractility of cultured myometrial cells according to the manufacturer’s instructions. Briefly, myometrial cells transfected with Sh-*Rac1* and Sh-NC transfection were harvested with trypsin and then resuspended in DMEM containing 10% FBS. Collagen solution (CBA-201, Cell Biolabs, CA, USA) was combined with cells to achieve a cell density of 1 × 10^5^ cells/mL and a final collagen concentration of 1.0 mg/mL. The cell–collagen mixture was pipetted into a 24-well plate and incubated for 1 h at 37°C to allow polymerization. After the cultures were incubated for 2 days in DMEM with 10% FBS, the gel was gently released and detached from the sides of the culture dishes immediately after changing DMEM containing 10% FBS and 100 ng/mL LPS. Twenty-four hours later, the gels were photographed, and the areas were measured using ImageJ Data Acquisition Software (NIH, Maryland, USA).

### Western blotting

Protein extraction was conducted using radioimmunoprecipitation assay (RIPA) buffer with a protease inhibitor (04693132001, Roche, San Cugat del Vallés, Barcelona, Spain). Then, the suspension was centrifuged (12,000 rpm for 10 min at 4°C), and the total protein content was measured with a BCA Protein Assay kit (23225, Thermo Fisher Scientific). The samples (20 µg/lane) were subjected to 12% SDS-PAGE and subsequently transferred to nitrocellulose membranes (Pall, NY, USA). After blocking with 5% non-fat milk in Tris-buffered saline (TBS)–Tween for 1 h at room temperature, the membranes were incubated overnight at 4°C with specific primary antibodies against RAC1 (1:2000, 05-389, Sigma–Aldrich), COX-2 (1:2000, 12282S, Cell Signalling), tubulin (1:5000, 200608, Zen-BioScience, Chengdu, China) and GAPDH (1:5000, 200306-7E4, Zen-BioScience, Chengdu, China). Then, the membranes were incubated with secondary antibodies (goat anti-mouse IgG H&L (HRP), 511103; goat anti-rabbit IgG H&L (HRP), 511203, Zen-BioScience, Chengdu, China) at a 1:5000 dilution for 2 h at room temperature. Next, chemiluminescent detection (Millipore) was used for signal analysis. Specific proteins were calculated using ImageJ Data Acquisition Software.

### RAC1 activity assay

Active RAC1 was pulled down by incubation lysates with GST-PAK-PBD (p21-activated kinase protein-binding domain) using the RAC1 activation assay kit (17-283, Sigma–Aldrich) according to the manufacturer’s instructions. Briefly, mouse myometrial tissues were homogenized with lysis buffer. The cell lysates (1 mg) were incubated with 10 μL GST-PAK-PBD beads for 1 h at 4°C. The beads were washed, suspended in 2 × Laemmli sample buffer (S3401-10VL, Sigma–Aldrich) and separated by SDS–PAGE. GTP-bound RAC1 levels were determined using a monoclonal mouse anti-RAC1 antibody (1:2000, 05-389, Sigma–Aldrich).

### Immunofluorescence

The paraffin-embedded sections were deparaffinized and rehydrated. Then, antigens were retrieved in 10 mM boiled citrate buffer (pH 6.0, Beijing Solarbio Science & Technology Co., Ltd., China) for 30 min. Following permeabilization with 0.3% Triton X-100 in PBS for 30 min, the sections were incubated with PBS containing 5% goat serum and 0.3% Triton X-100 for 2 h to block non-specific binding. To evaluate the expression of Rac1, the sections were incubated with mouse anti-RAC1 (1:200, 05-389, Sigma–Aldrich) overnight at 4°C, followed by incubation with FITC-conjugated goat anti-mouse IgG (H+L) cross-adsorbed secondary antibody (1:500, F-2761, Invitrogen, Thermo Fisher Scientific, Inc.) for 2 h at room temperature. After being washed with PBS, the nuclei were counterstained with 4′-DAPI (Sigma–Aldrich) for 5 min. The primary antibody was diluted with blocking buffer, and isotype control antibodies were used as a negative control (Mouse IgG Isotype Control, 10400C, Invitrogen, Thermo Fisher Scientific, Inc.). Images were captured with a Pannoramic MIDI digital microscope (3DHISTECH, Budapest, Hungary) and were analysed using CaseViewer (3DHISTECH, Budapest, Hungary).

### Statistical analysis

The data were analysed with GraphPad Prism 7 software (GraphPad). The data are presented as mean ± s.e.m. Histological evaluation was analysed using the Mann–Whitney *U* test. Comparisons between two groups were performed using unpaired Student’s *t* test. The percentage of preterm births was analysed with the chi-square test. Comparisons among multiple groups were analysed with one-way ANOVA, followed by Tukey’s test for multiple comparisons. Statistical significance was defined as *P* < 0.05.

## Results

### NSC23766 attenuates inflammation-induced preterm birth in mice

**Figure 1:**
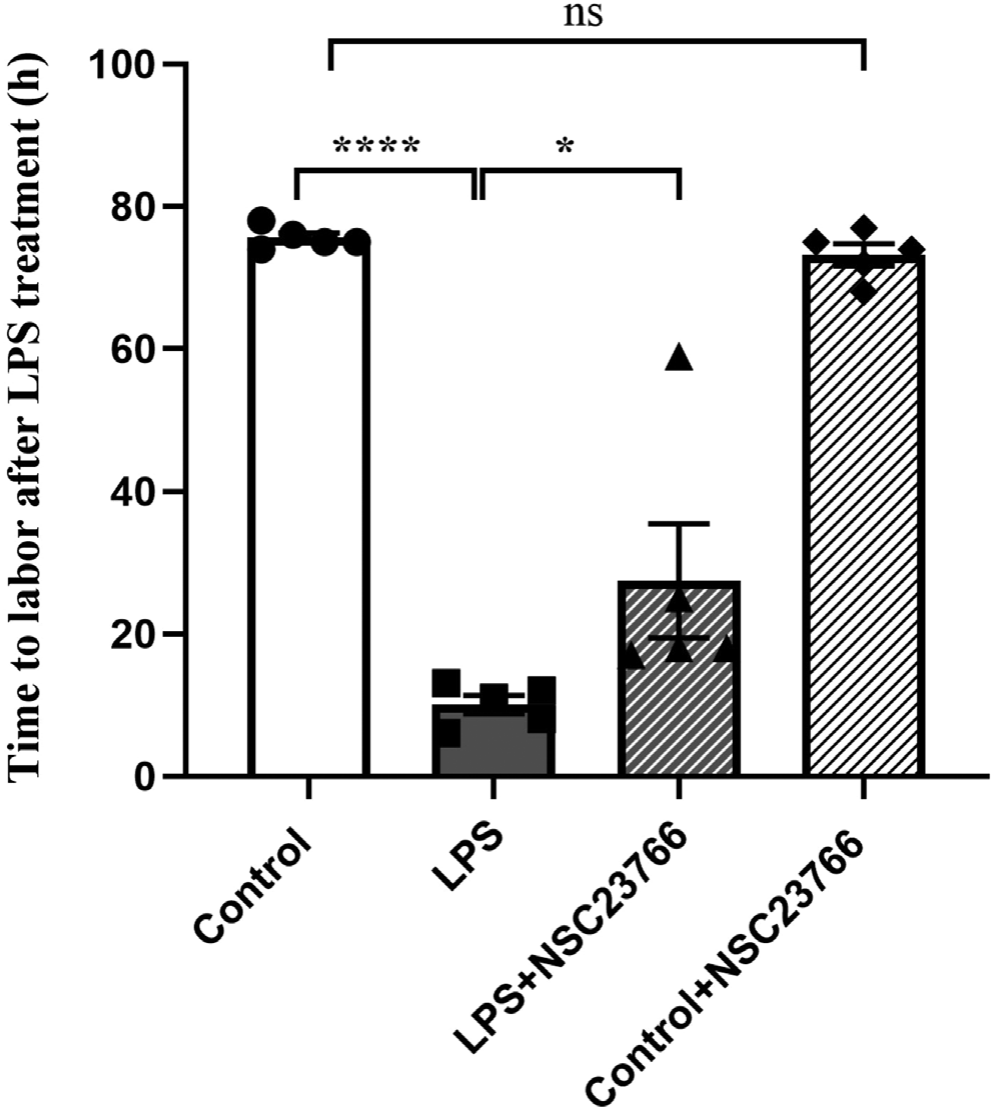
NSC23766 attenuates inflammation-induced preterm birth in mice. *n* = 5 mice per group. ^*^ *P* < 0.05, ^****^ *P* <0.0001, ns *P* > 0.05 one-way ANOVA, Tukey’s multiple comparisons test.

According to our preliminary experiment, preterm birth occurred within 6–12 h post-LPS administration. We compared the rate of preterm delivery within 12 h and pregnancy duration after LPS treatment. After LPS injection on GD16, 93.8% of pregnant mice experienced pregnancy failure within 12 h, and the pregnancy duration from LPS treatment was 10.0 ± 1.3 h (Fig. 1). NSC23766 pretreatment reduced the rate of pregnancy failure from 93.8 to 40% within 12 h (data shown in Table 1) and delayed the onset of LPS-induced preterm birth in mice (Fig. 1). There was no difference between control animals with or without NSC23766 (75.6 ± 0.6 h vs 73.2 ± 1.3 h).

**Table 1:** NSC23766 attenuates inflammation-induced preterm birth.

Groups	Preterm births,* n*	Pregnant mice, *n*	Preterm delivery rate, %
Control	1	22	4.5
LPS	15	16	93.8^*^
LPS + NSC23766	6	15	40^#^
Control + NSC23766	1	15	6.2

^*^P < 0.05 vs control; ^#^*P* < 0.05 vs LPS, chi-square test.

### Intrauterine infusion of LPS caused a decline in progesterone levels and enhanced spontaneous uterine myometrium contraction *in vitro*

**Figure 2:**
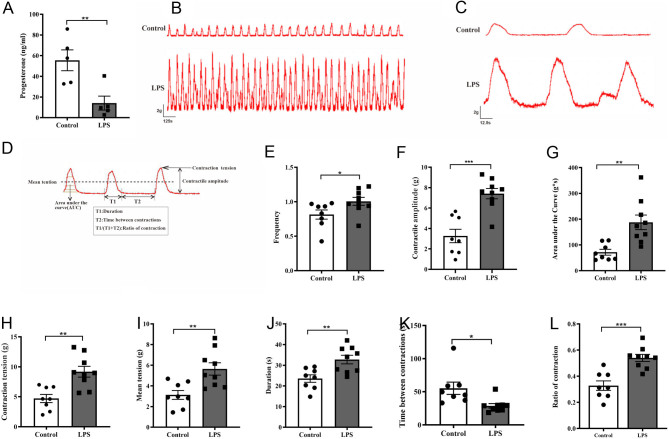
LPS elevates progesterone levels and promotes the contraction of uterine strips from pregnant mice. (A) Blood progesterone levels in mice were measured by ELISA, *n*  = 5 mice per group. ^**^ *P* < 0.01, Student’s *t* test. (B) Parameters reflecting a contraction of the uterine strips. (C) Representative recordings show spontaneous phasic contractions of uterine strips in the control and LPS groups. (D) Individual contractile events on a magnified time scale. Bar graphs show the frequency (E), contractile amplitude (F), area under the curve (G), contraction tension (H), mean tension (I), duration (J), time between contractions (K) and ratio of contraction (L) of spontaneous isometric contractions. Columns represent the mean ± s.e.m., *n*  = 8–9 mice per group, ^*^P < 0.05, ^**^P < 0.01 and ^***^P < 0.001, Student’s *t* test.

To investigate the LPS-induced changes, serum progesterone levels and* in vitro* spontaneous uterine myometrium contraction parameters were measured. The serum progesterone level was significantly diminished (*P* < 0.01 vs control) 12 h after LPS administration (Fig. 2A).

The parameters used to assess uterine myometrium contraction in our experiment are shown in Fig. 2D. In Fig. 2B and C, a representative recording of spontaneous contraction generated by a uterine strip was shown. Our results indicated that uterine strips showed significant contractile activity 12 h after LPS administration; there were especially notable increases in frequency (LPS 1.01 ± 0.06/min vs control 0.81 ± 0.07/min, *P* < 0.05), contractile amplitudes (LPS 7.03 ± 0.60 g vs control 3.27 ± 0.65 g, *P* < 0.001), area under the curve (LPS 187.60 ± 28.30 g*s vs control 71.83 ± 11.33 g*s, *P* < 0.01), contraction tension (LPS 9.18 ± 0.90 g vs control 4.71 ± 0.67 g, *P* < 0.01), mean tension (LPS 5.64 ± 0.59 g vs control 3.13 ± 0.43 g, *P* < 0.01), duration (LPS 32.72 ± 2.08 s vs control 23.56 ± 1.80 s, *P* < 0.01), time between contractions (LPS 28.82 ± 3.42 s vs control 55.37 ± 9.26 s, *P* < 0.05) and ratio of contraction (LPS 0.54 ± 0.03 vs control 0.34 ± 0.04, *P* < 0.001) (Fig. 2E, F, G, H, I, J, K and L).

### Changes in inflammatory cells in the myometrium

**Figure 3:**
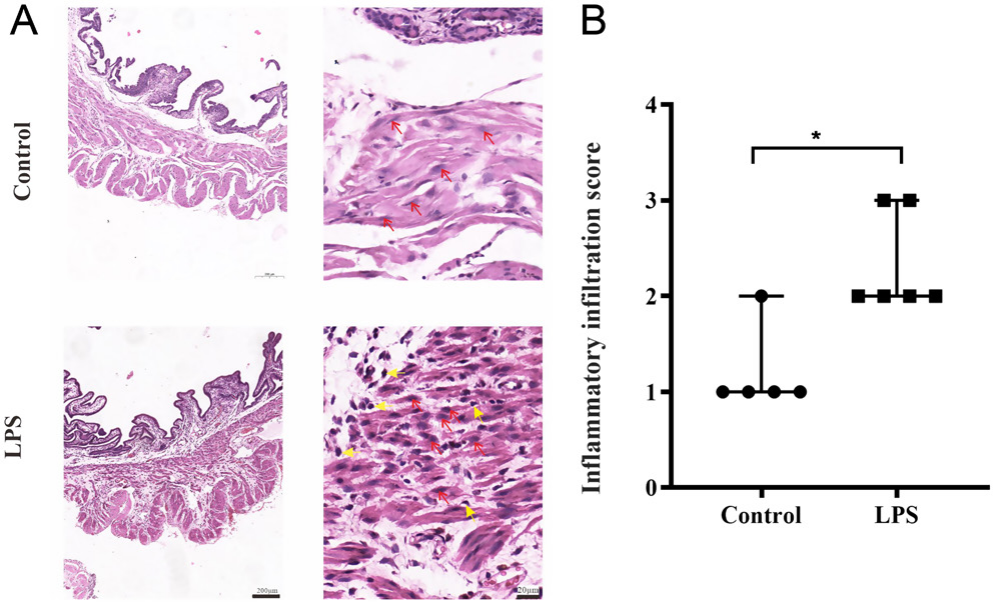
LPS causes morphological changes in mouse uteruses. (A) Representative histological H&E-stained micrographs of myometrial sections from the control and LPS groups. Inflammatory cells are indicated with yellow arrows, and smooth muscle cells are indicated with red arrows. (B) Morphological evaluation of myometrial inflammatory cell infiltration (*n*  = 5–6 mice per group). The severity of inflammatory cell infiltration was graded from 0 to 3, ^*^P <0.05, Mann–Whitney *U* test.

It has been hypothesized that parturition may be partly associated with the activation of proinflammatory pathways (Cappelletti *et al.* 2020). To examine the impact of LPS challenge on intrauterine inflammation in our mouse model, the severity of inflammatory cell infiltration in the myometrium was assessed by HE. Inflammatory cells were evident in the connective tissue between muscle bundles in both circular and longitudinal muscles (Fig. 3A). Histopathological severity grades were evaluated based on the extent of inflammatory cell infiltration. The inflammatory cell density appeared to be increased in the myometrium 12 h after LPS administration (Fig. 3B, P < 0.05 vs control). Our data showed that LPS administration promoted the influx of inflammatory cells, revealing that 150 µg/dam LPS led to myometrial inflammation to successfully induce preterm birth.

### LPS upregulates the expression of uterine myometrium contraction proteins in mice and RAC1 expression increases in human participants experiencing preterm delivery

**Figure 4:**
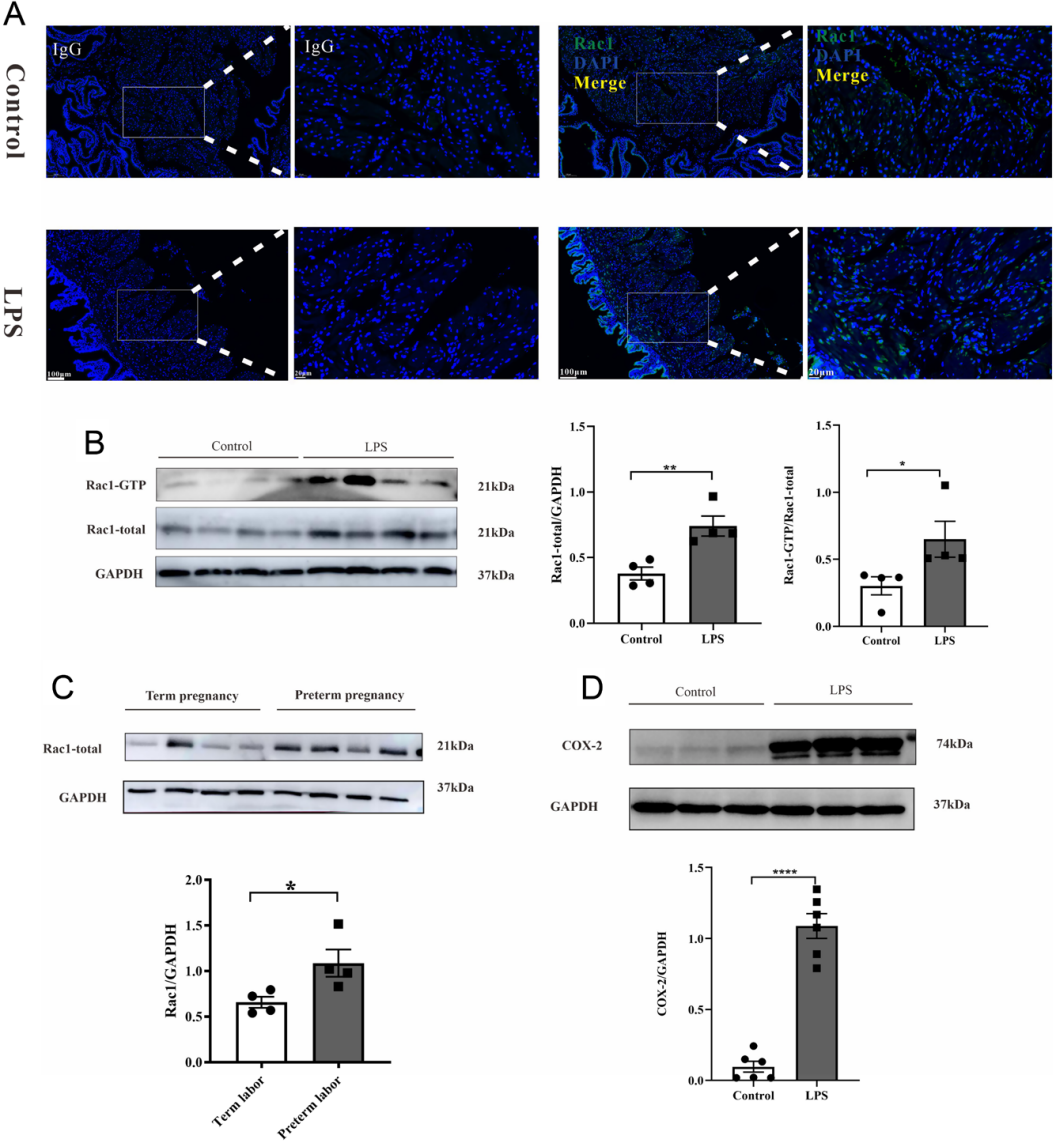
RAC1 protein expression is increased in mice and human uteri. (A) Representative RAC1-staining on myometrial sections of mice. (B) Representative Western blots showing mouse uterine RAC1 expression and activity and quantification analysis (*n*  = 4 mice per group). (C) Representative Western blots showing RAC1 expression in the human uterus in parturient participants who underwent caesarean section and quantification analysis (*n*  = 4 mice per group). (D) Representative Western blots showing mouse uterine COX-2 expression and quantification analysis (*n*  = 6 mice per group). Columns represent the mean ± s.e.m., ^*^P < 0.05, ^**^P < 0.01, ^****^P < 0.0001, Student’s *t* test.

As shown in Fig. 4A, RAC1 fluorescence was distributed in the cytoplasm of myometrial SMCs. It is plausible that LPS exposure induced significantly higher RAC1 fluorescence intensity than the control. Therefore, we next assessed RaAC1 protein expression in mice and human uteri via Western blotting analysis (Fig. 4B). Compared with that in the control mice, uterine RAC1 expression in the mice injected with LPS was substantially increased and almost doubled (*P* < 0.01, Fig. 4B). A pull‐down assay was conducted to examine RAC1 activation, and the results showed that LPS induced RAC1 activation (RAC1-GTP) (*P* < 0.05, Fig. 4B). We also measured RAC1 protein levels in the uterus of participants with preterm delivery to verify the relationship between RAC1 and preterm delivery. Consistent with that in mice, the data suggested that RAC1 expression was increased in the uterus of participants with premature delivery compared with that in the uterus of participants with term delivery (*P* < 0.05, Fig. 4C).

Cyclooxygenase (COX), the rate-limiting enzyme in prostaglandin synthesis, contributes to parturition. COX-2 protein levels further increase in pregnancy after exposure to inflammatory stimuli. The results showed that LPS exposure significantly upregulated the COX-2 protein levels by as much as six-fold in the myometrium compared to the control treatment (*P* < 0.0001, Fig. 4D).

### NSC23766 reduces spontaneous uterine myometrium contraction

**Figure 5:**
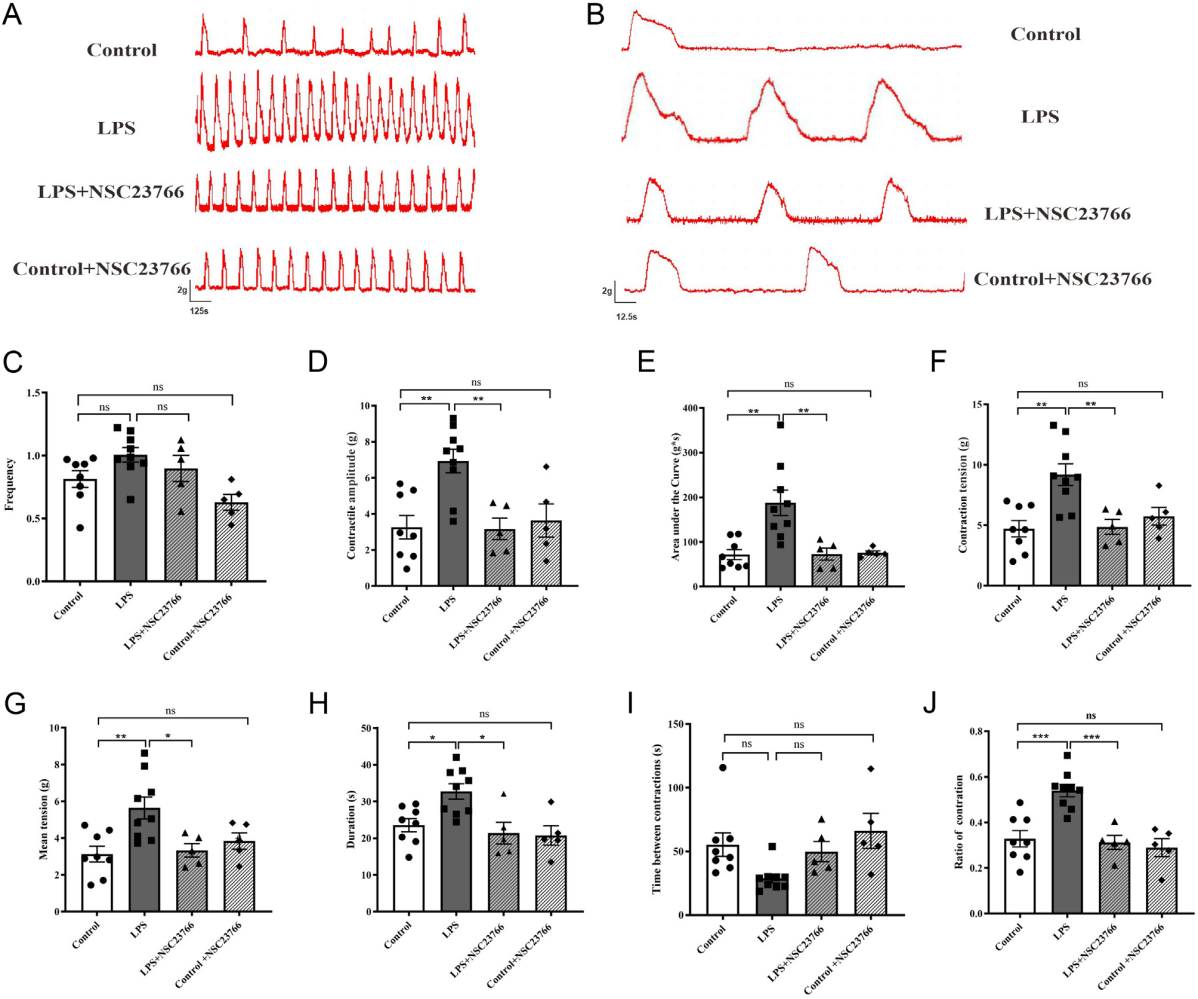
NSC23766 inhibits the contraction of uterine strips in pregnant mice. (A) Representative recordings show spontaneous phasic contractions of uterine strips from different groups. (B) Individual contractile events on a magnified time scale. Bar graphs show the frequency (C), contractile amplitude (D), area under the curve (E), contraction tension (F), mean tension (G), duration (H), time between contractions (I) and ratio of contraction (J) of spontaneous isometric contractions. Columns represent the mean ± s.e.m., *n*  = 5–9 mice per group, ^*^P < 0.05, ^**^P < 0.01, ^***^P < 0.001, ns *P* > 0.05, one-way ANOVA, Tukey’s multiple comparisons test. The data of the control and LPS groups are the same as in Fig. 2.

We next further examined whether RAC1 regulates uterine myometrium contraction and affects premature delivery. The uterine contractility records (Fig. 5A and B) indicated that the spontaneous contraction of uterine muscle strips from the LPS group treated with NSC23766 was significantly decreased but that of uterine muscle strips from mice treated with saline remained unchanged. NSC23766 exerted an inhibitory effect (Fig. 5C, D, E, F, G, H, I and J) on LPS-increased contractile amplitudes (3.17 ± 0.60 g vs LPS 6.94 ± 0.65 g, *P* < 0.01), area under the curve (72.88 ± 13.43 g*s vs LPS 187.6 ± 28.3 g*s, *P* < 0.01), contraction tension (4.87 ± 0.62 g vs LPS 9.18 ± 0.90 g, *P* < 0.01), mean tension (3.33 ± 0.37 g vs LPS 5.64 ± 0.59 g, *P* < 0.05), duration (21.39 ± 2.96 s vs LPS 32.72 ± 22.08 s, *P* < 0.05) and the ratio of contraction (0.31 ± 0.03 vs LPS 0.54 ± 0.03, *P* < 0.001) but did not alter the frequency or time between contractions. We also observed a nearly 30% increase in the time between contractions after NSC23766 treatment, but the difference was not statistically significant (*P* > 0.05 vs LPS). These data indicated that uterine myometrium contraction in LPS-exposed mice is mediated by Rac1 signalling.

### NSC23766 reduced RAC1 activation and COX-2 expression

**Figure 6:**
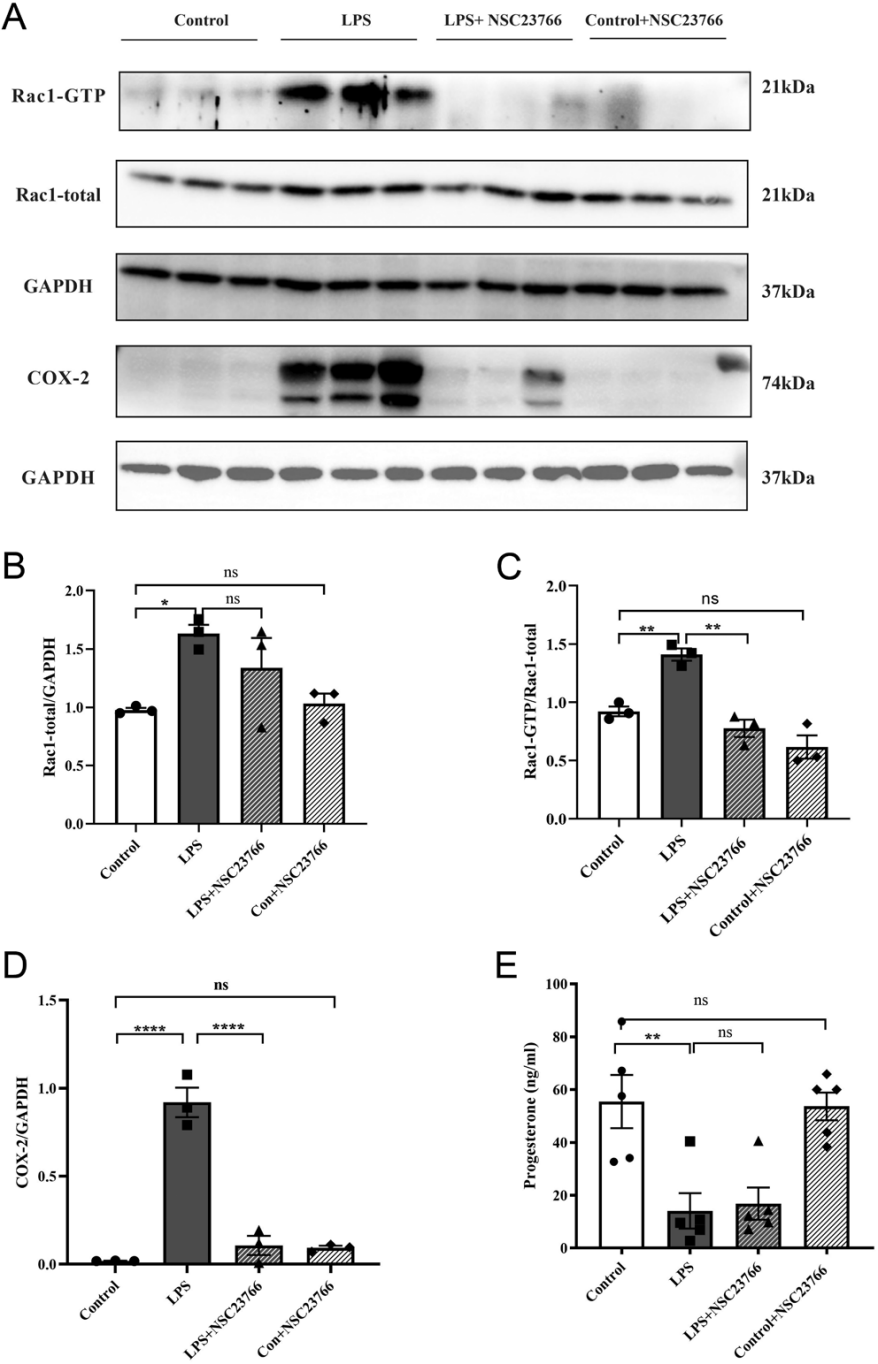
NSC23766 affects RAC1 activity and COX-2 protein expression and progesterone levels. (A) Representative Western blots showing RAC1 alteration and COX-2 expression in the mouse uteruses. (B-D) Graphs showing the mean band density from the Western blots in A (*n*  = 3 mice per group). (E) NSC23766 had no effect on the progesterone levels (*n*  = 5 mice per group, the data of the control and LPS groups are the same as in Fig. 2). Columns represent the mean ± s.e.m., ^*^P < 0.05, ^***^P < 0.001, ^****^P < 0.0001, ns *P* > 0.05, one-way ANOVA, Tukey’s multiple comparisons test.

Following the inhibition of RAC1, Western blotting assay results indicated that compared with LPS, NSC23766 did not affect RAC1 protein levels (*P* > 0.05, Fig. 6A and B). It is known that NSC23766 inhibits RAC1 activation but not RAC1 expression. Therefore, we examined RAC1 activation in myometrial tissue. LPS-induced RAC1 activation was inhibited by the application of NSC23766 (*P* < 0.01, Fig. 6A and C). NSC23766 inhibited the protein level of COX-2 compared to the LPS group (*P* < 0.0001, Fig. 6A and D). The serum progesterone level after LPS was unaffected by NSC23766 (*P* > 0.05, Fig. 6E). In summary, RAC1 upregulation and activation may have a significant effect on COX-2.

### Silencing of *Rac1* impaired contractility and downregulated COX-2 expression in cultured myometrial cells

**Figure 7:**
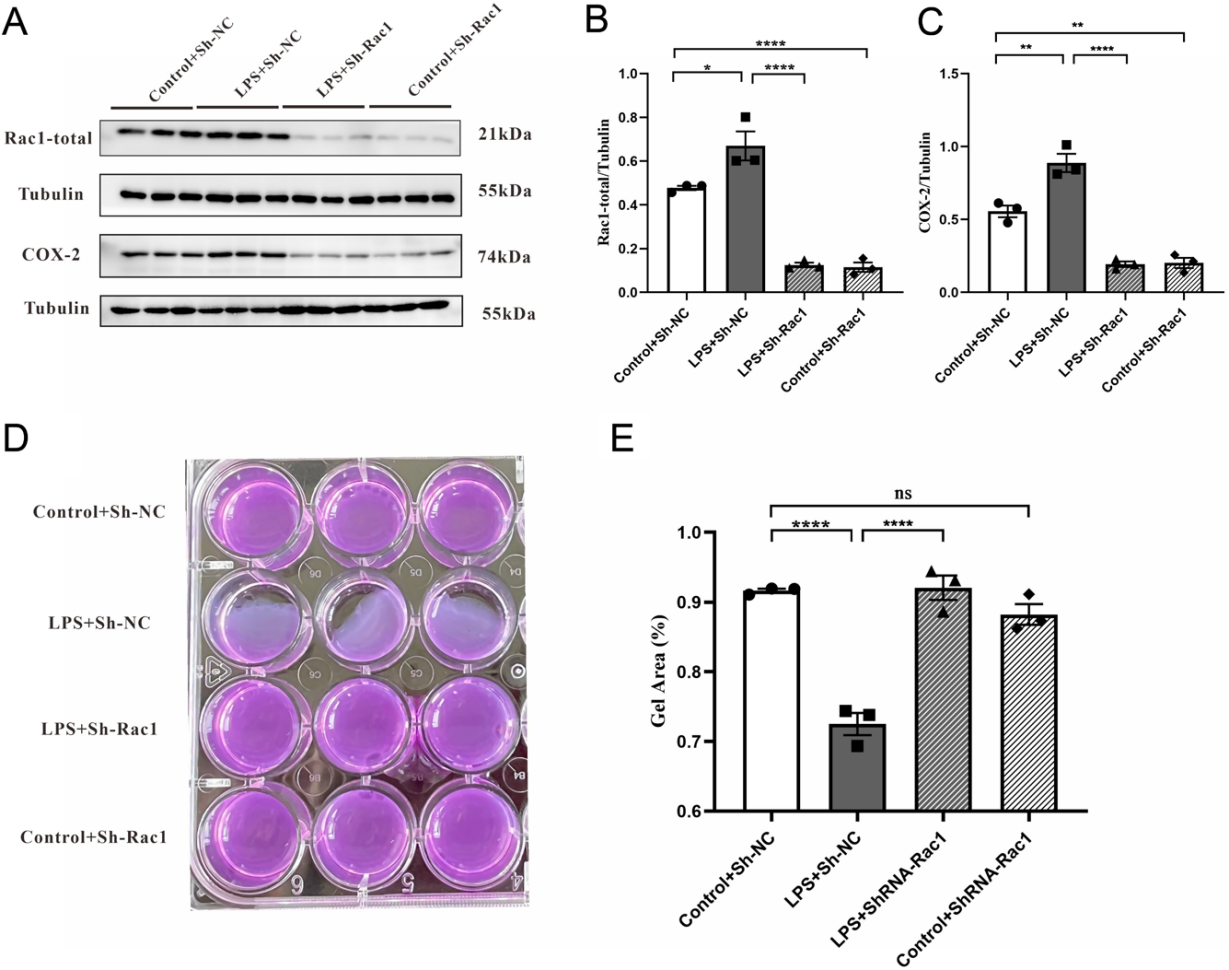
*Rac1* promotes contractility and COX-2 expression in cultured mouse USMCs. (A) Representative Western blots showing *Rac1* and COX-2 expression in cells transfected with Sh-*Rac1*. (B-C) Graphs showing the mean band density from the western blots in A. (D-E) Effect of *Rac1* knockdown on contractility of USMCs. The contractility was determined by collagen contractility assay. Columns represent the mean ± s.e.m., *n*  = 3 per group, ^*^P < 0.05, ^**^P < 0.01, ^****^P < 0.0001, ns *P* > 0.05, one-way ANOVA, Tukey’s multiple comparisons test.

LPS treatment significantly elevated *Rac1* and COX-2 expression compared with the control (Fig. 7A). The knockdown efficiency of Sh-*Rac1* transfection was confirmed by Western blotting, and Sh-*Rac1* led to an approximately 80% reduction in *Rac1* expression (*P* < 0.0001, Fig. 7A and B). Interestingly, we also observed that treatment of cells with LPS and Sh-*Rac1* reduced COX-2 expression compared with vehicle treatment (*P* < 0.0001, Fig. 7A and C). Additionally, we observed the effects of *Rac1* knockdown on contraction. As expected, the cells treated with LPS and transfected with Sh-*Rac1* displayed less contractility than Sh-NC, as evidenced by the collagen area (*P* < 0.0001, Fig. 7D and E). This result was consistent with treatment with NSC23766* in vivo*. Overall, the data showed that *Rac1* was involved in contractility and modulated COX-2 expression.

## Discussion

In the present study, our data clearly indicate that LPS-induced preterm delivery is associated with the regulation of RAC1. Using a mouse model of premature delivery, we observed an increase in myometrial contractility and *Rac1* expression activation. In particular, NSC23766 treatment suppressed myometrial contractility and COX-2 protein levels, indicating that RAC1 exerts certain regulatory effects on myometrial contraction in inflammation-mediated PTB. Although it has been suggested that the mechanism underlying uterine myometrium contraction is complex, our results demonstrate, for the first time, that RAC1 plays a critical role in myometrial contraction and may have direct translational implications in the treatment of preterm birth.

PTB causes substantial public health issues due to associated complications, such as neurocognitive, behavioural and motor disabilities (Lindström *et al.* 2009). It has been reported that intrauterine inflammation is related to premature delivery, accounting for up to 40% of cases (Agrawal & Hirsch 2012). In the literature, animal models of intrauterine LPS administration are well established and used to elucidate the mechanisms underlying fetal brain injury caused by preterm birth (Burd *et al.* 2009, 2010, Elovitz *et al.* 2011, Dambaeva *et al.* 2018). The animal model can simulate human intrauterine infection conditions. Hence, to investigate the mechanisms underlying preterm birth, our study used LPS exposure to induce intrauterine inflammation.

Although we used an inflammatory PTB mouse model, inflammatory cytokines, such as IL-1, IL-6 or TNF alpha (TNFα), were not examined in the present study. Inflammatory stimuli produce cytokines and chemokines to stimulate the recruitment of peripheral leukocytes into gestational tissues, which is the mechanism by which the inflammatory cascade leads to preterm birth (Olson *et al.* 2015). Leukocytes, including macrophages, neutrophils and monocytes, invade the myometrium and produce cytokines, amplifying the inflammatory cascade. Previously, Thomson *et al.* showed that LPS-induced preterm labour was indeed associated with increased influx of inflammatory cell into the myometrium (Thomson *et al.* 1999). Similarly, in our current study, the H&E staining result showed that the degree of inflammatory cell infiltration in the myometrium in response to LPS was higher than that in the control, indicating that the model in our study was successful.

The precise mechanism of infectious PTB is complex, but the final pathway is the induction of uterine myometrium contraction. Therefore, uterine myometrium contraction is thought to be an objective indicator of PTB and holds great clinical significance. In PTB physiology, myometrial contraction is abnormal. In our LPS-induced PTB mouse model, we found that multiple variables, such as the frequency, contractile amplitudes, area under the curve, contraction tension, mean tension, contraction duration and ratio of contraction, increased. This result was similar to the effect of oxytocin on the contraction of myometrial strips (Edwards *et al.* 1986). Notably, the enhancement of myometrial contraction is stimulated by bacterial LPS, which agrees with a previous study (Ge *et al.* 2018). LPS stimulates TLR4 signalling, enhancing inflammatory and immune responses (Cappelletti *et al.* 2020). Notably, LPS exposure modulates the contractility of vascular smooth muscle cells by increasing IL-6 secretion (Strela *et al.* 2020). However, in addition to the increased production of inflammatory cytokines, including TNFα, IL-6 and IL-22, in response to LPS (Gomez-Lopez *et al.* 2010, Wakabayashi *et al.* 2013, Dambaeva *et al.* 2018), other pathways may be involved in LPS-induced myometrial contraction. The mechanism involved in this phenomenon remains to be elucidated.

Progesterone plays an essential role in maintaining pregnancy primarily by promoting uterine quiescence. In the present study, LPS treatment resulted in a reduction in progesterone in maternal circulation. Increasing evidence has demonstrated that pretreatment with progesterone is associated with the suppression of inflammatory mediators after LPS exposure (Elovitz & Wang 2004, Shields *et al.* 2005). Recently, much attention has been given to the LPS-induced activation of *Rac1* (Yin *et al.* 2017, Liu *et al.* 2020). There is accumulating evidence that RAC1 can stimulate NF-κB activity to enhance microglial inflammatory activity (Bianchi *et al.* 2010) and upregulate COX-2 expression in recurrent respiratory papillomas (Wu *et al.* 2010, Liu *et al.* 2020). In our current study, we used NSC23766 to determine the effect of RAC1 on the production of COX-2. We found that the COX-2 level was upregulated in response to LPS. NSC23766 decreased the expression of COX-2, indicating that RAC1 regulated COX-2.

To the best of our knowledge, no research has been conducted on the effect of RAC1 on progesterone, so we speculated whether there was a link between RAC1 and progesterone. Our results showed that *Rac1* inhibition had no effect on the levels of progesterone. Therefore, there should be mechanistic overlap between infections and the production of uterine myometrium contraction to cause preterm birth. RAC1 has been shown to mediate SMC contraction (Sakai *et al.* 2018) and proliferation (Yin *et al.* 2017, Andre-Gregoire *et al.* 2018). It was demonstrated that the activation of RAC1 modulated phospholipase (PLC) β2 to upregulate intracellular Ca^2+^ signalling and ultimately enhanced contraction (Kai *et al.* 2019). We speculate that RAC1 is involved in the regulation of myometrial contraction via the modulation of intracellular Ca^2+^ concentrations. In the present study, *Rac1* expression in uterine samples from mice was measured 12 h after LPS administration. However, without LPS treatment, there was no difference in the protein levels of RAC1 in the uterus on different gestational days (day 7, day 16 and day 19) (data shown in Supplementary Fig. 2). The present data revealed that RAC1 expression was significantly induced by LPS, not gestational day. To our knowledge, RAC1 is an essential process for inflammation activation and may play a role in maternal immune tolerance. Compared to control animals, we found that all LPS-treated animals exhibited increased uterine RAC1 expression and activity. In addition, compared with that in participants with term delivery, we found that the expression of uterine RAC1 in participants with PTB was significantly higher.

In addition, the role of RAC1 in pregnant myometrial contraction can be verified in contractility studies by applying its inhibitor, NSC23766. We found that NSC23766 inhibited contraction in our study, similar to its effect on the contraction of the mouse urinary bladder and saphenous artery (Rahman *et al.* 2014). Our results demonstrated that treatment with NSC23766 significantly reduced the onset of labour within 12 h and prolonged the pregnancy duration after injection of LPS. It is plausible that NSC23766 prevents LPS-induced preterm birth. Moreover, treatment with NSC23766 inhibited RAC1 activity and simultaneously suppressed LPS-induced increases in uterine contraction and inflammatory maker expression (COX-2). Together, it appears possible that RAC1 activation could be related to preterm birth and would be responsible for the enhanced myometrial contraction and increased COX-2 expression.

Moreover, *Rac1* was knocked down by transfection with specific shRNAs to explore its role* in vitro* in the present study. We confirmed that *Rac1* knockdown reduced cell contractility and expression levels of COX-2 in cells treated with LPS. In other words, silencing *Rac1* ameliorated the LPS-mediated pathological changes in mouse USMCs, which was consistent with treatment with NSC23766* in vivo*. Together, these data might indicate that *Rac1* regulates uterine myometrium contraction and plays a key role in the pathological alterations associated with inflammation-mediated preterm birth. From the present data, we suggested that *Rac1* expression or RAC1 activity was implicated in uterine activation for labour.

There are several limitations in the present study. First, we only enrolled four term pregnant women and four preterm pregnant women in our study. To better understand the role of RAC1 in uterine myometrium contraction in human, large clinical trials may be needed in the future. Secondly, NSC23766 was employed as a RAC1 inhibitor to identify the contribution of RAC1 in the present study. While NSC23766 is a widely used inhibitor of RAC1 activation, it is worth mentioning that NSC23766 is a blocker of RAC1 interaction with its GEFs, Tiam1 and Trio rather than a direct inhibitor of RAC1 activity (Gao *et al.* 2004). Thirdly, COX-2 is associated with uterine inflammatory reaction. Although we found that *Rac1* inhibition decreased the expression levels of COX-2 protein, future studies could address their potential functional interactions and specific mechanism in modulating myometrial contraction. Fourthly, the roles of uterine RAC1 in uterine myometrium contraction require to be further confirmed by *Rac1* gene overexpression in uterine smooth muscle cells.

In summary, we provide convincing evidence that RAC1 contributes to uterine myometrium contraction. We found that LPS activated *Rac1* to enhance myometrial contraction, which is associated with preterm birth and parturition. However, the specific signalling pathways by which RAC1 participates in myometrial contraction are unclear, and the mechanisms need to be characterized with greater precision.

## Conclusion

In conclusion, our results reveal a new role of RAC1 in enhancing myometrial contraction in the pathogenesis of infection-associated PTB. RAC1 inhibition was able to reduce the muscle tension of the uterus. Therefore, inhibiting RAC1 expression and activity could provide a new strategy for the treatment of preterm birth characterized by inflammation.

## Supplementary Material

Supplementary Figure 1. A schematic diagram of the experimental model

Supplementary Figure 2. The expression level of Rac1 remains unchanged in different gestation days

## Declaration of interest

The authors declare that there is no conflict of interest that could be perceived as prejudicing the impartiality of the research reported.

## Funding

The study was supported by the National Natural Science Foundation of China (No. 81871192 to X M L).

## Author contribution statement

Min Diao conducted the study, extracted the data, performed the data analysis and wrote the manuscript. Jin Zhou helped in recording spontaneous uterine myometrium contraction. Yunkai Tao established the mouse model and collected tissue. Xuemei Lin designed the study, supervised the work and reviewed and edited the manuscript. Zhaoyang Hu designed the study, supervised the work, reviewed and edited the manuscript.
