# Supplementary figures and images for: RAC1 is involved in uterine myometrium contraction in the inflammation-associated preterm birth
Source: Reproduction. 2022 Aug 26;164(4):169–81. doi: 10.1530/REP-21-0186 (PMC9513643; doi:10.1530/REP-21-0186)

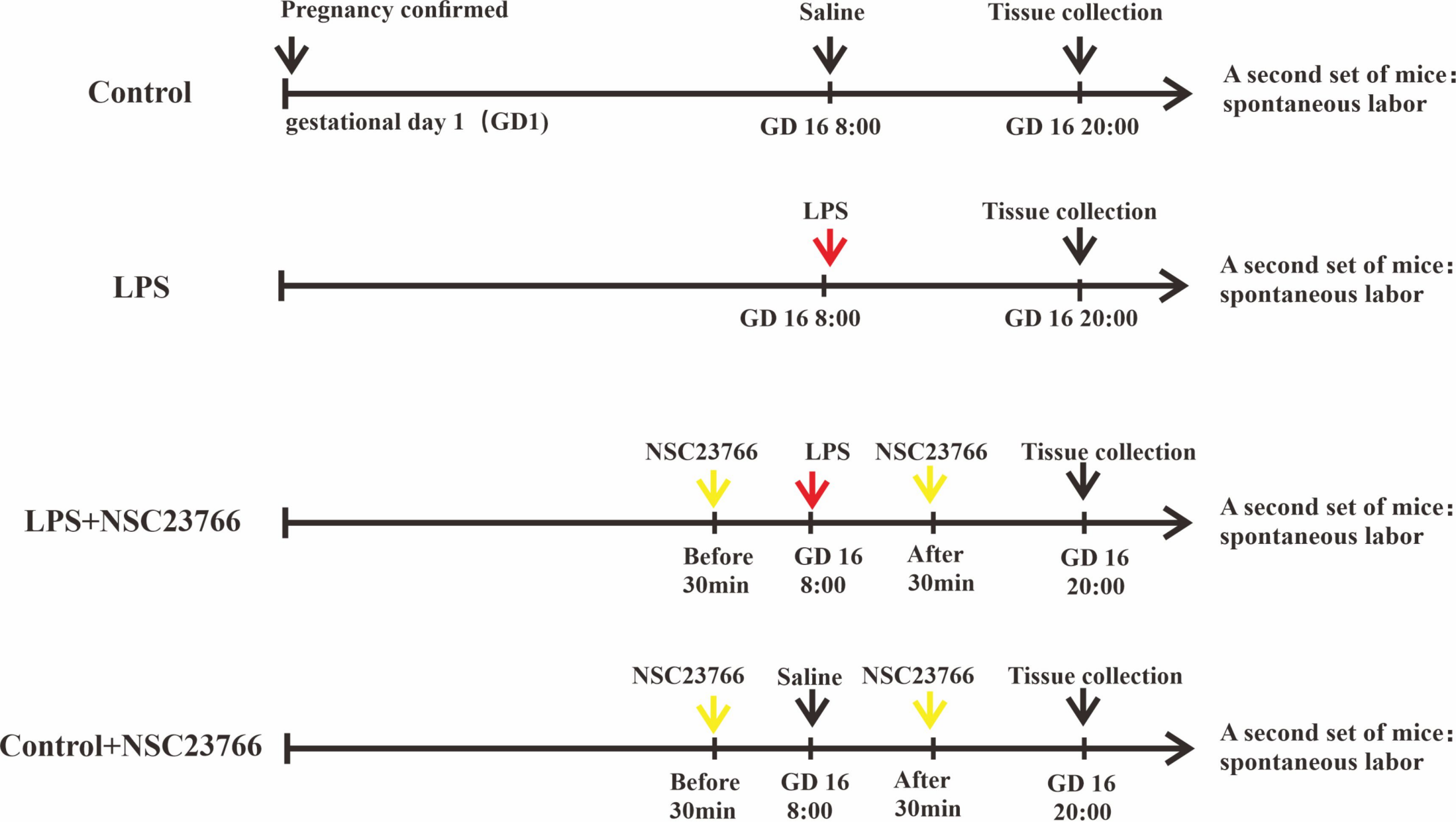

**Supplementary Figure 1. A schematic diagram of the experimental model**

Supplement: Supplementary Figure 1. A schematic diagram of the experimental model [file supplementary_figure_1.pdf]
