# Supplementary material for: RAC1 is involved in uterine myometrium contraction in the inflammation-associated preterm birth
Source: Reproduction. 2022 Aug 26;164(4):169–81. doi: 10.1530/REP-21-0186 (PMC9513643; doi:10.1530/REP-21-0186)
Supplement: Supplementary Figure 2. The expression level of Rac1 remains unchanged in different gestation days [file supplementary_figure_2.pdf]

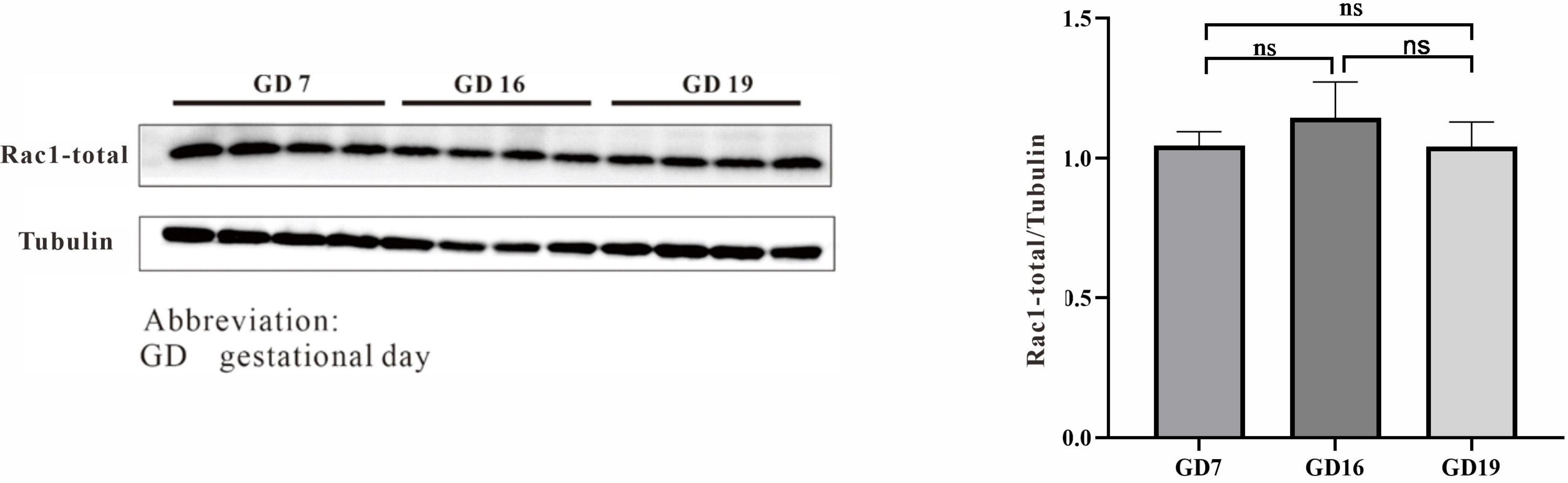

Supplementary Figure 2. The expression level of Rac1 remains unchanged in different gestation days
